# β-Aminopropionitrile-Induced Reduction in Enzymatic Crosslinking Causes *In Vitro* Changes in Collagen Morphology and Molecular Composition

**DOI:** 10.1371/journal.pone.0166392

**Published:** 2016-11-09

**Authors:** Silvia P. Canelón, Joseph M. Wallace

**Affiliations:** 1 Weldon School of Biomedical Engineering, Purdue University, West Lafayette, Indiana, United States of America; 2 Department of Biomedical Engineering, Indiana University-Purdue University at Indianapolis, Indianapolis, Indiana, United States of America; 3 Department of Orthopaedic Surgery, Indiana University School of Medicine, Indianapolis, Indiana, United States of America; Dalhousie University, CANADA

## Abstract

Type I collagen morphology can be characterized using fibril D-spacing, a metric which describes the periodicity of repeating bands of gap and overlap regions of collagen molecules arranged into collagen fibrils. This fibrillar structure is stabilized by enzymatic crosslinks initiated by lysyl oxidase (LOX), a step which can be disrupted using β-aminopropionitrile (BAPN). Murine *in vivo* studies have confirmed effects of BAPN on collagen nanostructure and the objective of this study was to evaluate the mechanism of these effects *in vitro* by measuring D-spacing, evaluating the ratio of mature to immature crosslinks, and quantifying gene expression of type I collagen and LOX. Osteoblasts were cultured in complete media, and differentiated using ascorbic acid, in the presence or absence of 0.25mM BAPN-fumarate. The matrix produced was imaged using atomic force microscopy (AFM) and 2D Fast Fourier transforms were performed to extract D-spacing from individual fibrils. The experiment was repeated for quantitative reverse transcription polymerase chain reaction (qRT-PCR) and Fourier Transform infrared spectroscopy (FTIR) analyses. The D-spacing distribution of collagen produced in the presence of BAPN was shifted toward higher D-spacing values, indicating BAPN affects the morphology of collagen produced *in vitro*, supporting aforementioned *in vivo* experiments. In contrast, no difference in gene expression was found for any target gene, suggesting LOX inhibition does not upregulate the LOX gene to compensate for the reduction in aldehyde formation, or regulate expression of genes encoding type I collagen. Finally, the mature to immature crosslink ratio decreased with BAPN treatment and was linked to a reduction in peak percent area of mature crosslink hydroxylysylpyridinoline (HP). In conclusion, *in vitro* treatment of osteoblasts with low levels of BAPN did not induce changes in genes encoding LOX or type I collagen, but led to an increase in collagen D-spacing as well as a decrease in mature crosslinks.

## Introduction

Bone is a composite material made up of an inorganic (hydroxyapatite mineral) phase, a proteinaceous organic phase, and water. Comprising 90% of bone’s organic phase, type I collagen is the most abundant protein in the human body [1]. Both hydroxyapatite and collagen contribute to bone mechanical properties; hydroxyapatite provides compressive strength and stiffness while collagen provides tensile strength and ductility [2–4]. Because bone is a hierarchical material, changes in the properties of either phase can influence bulk mechanical properties of the tissue and bone structure. In some cases, these effects can compromise bone’s ability to serve its structural function of bearing dynamic loads associated with movement. For example, decreased bone strength is a characteristic of osteoporosis and reflects deterioration in bone density and bone quality [5–7]. Osteogenesis imperfecta is also characterized by decreased bone strength and toughness, and is caused by disruptions in the quality or amount of type I collagen [8–10].

**Fig 1:**
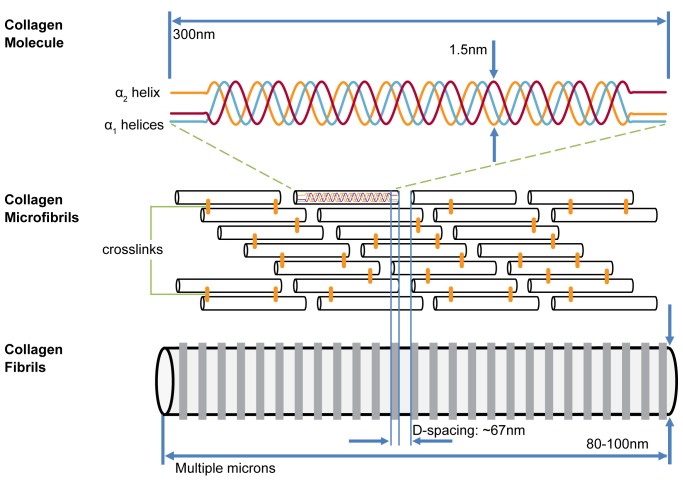
Collagen structure and organization. Collagen molecules self-assemble in a quarter-staggered array into microfibrils to form collagen fibrils with characteristic periodic D-spacing.

Type I collagen in bone is synthesized by mature osteoblasts as a right-handed helical structure formed from three polypeptide chains of amino acids. Each chain is a left-handed helix with repeating Gly-X-Y triplets where Gly is glycine, X is usually proline, and Y hydroxyproline [11,12]. In type I collagen molecules, two of these polypeptide chains are α1 helices and one is an α2 helix. Once a triple-helical molecule forms, N and C terminal ends are cleaved by proteinases, leaving mature collagen molecules. These molecules self-assemble in line with one another into microfibrils, then in parallel into quarter-staggered arrays with overlap and gap regions, and finally into three-dimensional fibrils. The overlap and gap regions produce an oscillating surface topography of axially repeating bands along the fibril length, referred to as the D-spacing or periodicity of the fibril [13] (Fig 1). This D-spacing is a morphometric characteristic of collagen fibrils and exists as a distribution of values near the theoretical 67 nm[14]. Changes in mean D-spacing or its distribution of values can be used to detect differences in collagen structure, tissue origin, and hydration state [15–19].

Post-translationally, collagen fibrils are stabilized within their staggered array by intramolecular and intermolecular crosslinks [20–22]. Enzymatic crosslink formation begins when telopeptide lysine and hydroxylysine precursors, through lysyl oxidase (LOX) initiation, convert to telopeptide aldehydes, allysine and hydroxyallysine, respectively [21,23]. The allysines, in combination with other precursors (i.e. helical lysine or hydroxylysine) form covalent chemical crosslinks. Some of these crosslinks mature to their trivalent form as pyridinolines and pyrroles. Crosslink synthesis can be limited by compounds such as penicillamine and β-aminopropionitrile (BAPN), resulting in a crosslink deficiency which characterizes a disease known as lathyrism [24–26]. BAPN is found in the seeds of the *lathyrus odoratus* plant, grown as a famine crop, and acts by irreversibly binding to the LOX active site. This binding prevents LOX from catalyzing aldehyde formation and subsequently blocks the formation of new crosslinks and the maturation of pre-existing immature crosslinks.

While BAPN has been shown to affect macroscale bone properties and nanoscale properties of type I collagen *in vivo* in rabbit, rat, and mouse models [27–29], knowledge of its direct effects on the morphology, expression, and crosslinking of collagen produced *in vitro* by osteoblasts is limited [30,31]. Evidence in the literature shows LOX mRNA expression increases with low levels of BAPN exposure, and decreases at higher concentrations as the differentiation process is impaired [31–33]. At low concentrations, as bioavailability of LOX is decreased, the cells may possess the ability to upregulate *LOX* expression to bring the level of available LOX back within a normal range. Few studies have investigated BAPN inhibition of LOX to block crosslink formation *in vitro* [30–32], and fewer still specifically attribute the effect to a change in immature or mature crosslinks [31]. The purpose of this study was to modify a key step in post-translational collagen synthesis to observe alteration to type I collagen in its native state. It was hypothesized that BAPN-induced inhibition of collagen crosslinking would (1) alter the D-spacing morphology of collagen produced *in vitro* by osteoblasts (2) drive upregulation of the LOX gene to compensate for the reduction in aldehyde formation, leading to an observed increase in mRNA expression and (3) inhibit the formation of mature crosslinks, specifically hydroxylysylpyridinoline.

## Materials and Methods

### Cell culture

MC3T3-E1 Subclone 4 (ATCC CRL-2593) murine preosteoblasts were obtained from the American Type Culture Collection (ATCC, Manassas, VA) and cultured in proliferation medium composed of α minimal essential medium (α-MEM, Life Technologies, Carlsbad, CA), 10% fetal bovine serum (FBS, GIBCO, Carlsbad, CA), 0.5% penicillin/streptomycin (GIBCO, Carlsbad, CA), and 1% L-glutamine (Hyclone, Logan, UT). MC3T3-E1 differentiation control medium consisted of proliferation medium supplemented with 50 μg/ml ascorbic acid (Thermo Fisher Scientific,Waltham, MA) and differentiation experimental medium was additionally supplemented with 0.25 mM BAPN-fumarate (Sigma Aldrich, St. Louis, MO) for crosslink inhibition experiments.

### Collagen synthesis for analysis of collagen morphology

MC3T3-E1 cells were cultured in T75 flasks and allowed to proliferate for 3 days until reaching 80% confluence. Once confluent, the cells were seeded into 60 mm dishes at a density of 500,000 cells per dish. Cells were seeded into eight dishes, four of them control dishes without BAPN and four treatment dishes with 0.25mM BAPN-fumarate. After seeding, the medium was replaced and supplemented with ascorbic acid for 14 days to allow differentiation and promote collagen synthesis.

### Atomic force microscopy (AFM)

Following 14 days of differentiation, the cultures were rinsed with phosphate-buffered saline (PBS) and treated with 10 mM ethylenediaminetetraacetic acid (EDTA, Life Technologies, Carlsbad, CA). Experiments in bone and tendon have shown that EDTA has negligible effects on collagen fibril morphology (data not shown) and was here used to encourage cellular detachment from the extracellular matrix (ECM) in order to expose the newly synthesized collagen matrix for AFM imaging. After treatment with EDTA, the matrix was rinsed with ultrapurified water (Milli-Q, EMD Millipore, Darmstadt, Germany) and allowed to dry. Five locations within each dish were imaged with a Bioscope Catalyst Atomic Force Microscope (Bruker, Santa Barbara, CA) in peak force tapping mode using ScanAsyst Fluid+ probes (Bruker, Santa Barbara, CA). Within each location, a 30 μm x 30 μm scan was performed to find areas where collagen was exposed by treatment with EDTA. A 10 μm x 10 μm scan was then performed to find suitable areas for closer examination followed by a final 3.5 μm x 3.5 μm scan of collagen fibrils appropriate for analysis. A 2 Dimensional Fast Fourier Transform (2D-FFT) was performed to extract D-spacing from approximately 10 individual collagen fibrils at each location, as previously described [16,34]. D-spacing analysis was performed on a minimum of 50 fibrils per dish and 200 fibrils per experimental group.

### Quantitative Reverse Transcription Polymerase Chain Reaction

An additional set of experiments was run for gene expression analysis by qRT-PCR. Cells from a single flask were seeded into ten dishes (five control and five supplemented with 0.25mM BAPN-fumarate). At the end of a 7 day differentiation period, the medium was removed from each culture dish and replaced with 1mL of TRIzol reagent (Invitrogen, CA). RNA isolation was performed using TRIzol reagent and reverse transcription (RT) was carried out using a High Capacity cDNA Reverse Transcription Kit (Life Technologies, Carlsbad, CA). PCR was performed using an ABI 7500 Fast PCR machine under the 9600 Emulation thermal cycling mode with SYBR Green primers and Power SYBR Green PCR master mix (Life Technologies, Carlsbad, CA). Primers were chosen for target genes encoding type I collagen α1 (*COL1A1*), type I collagen α2 (*COL1A2*) and *LOX* as well as reference gene β-actin (*BACT*) [35,36]. Each sample/gene combination was run in triplicate and water was used as the no-template control. mRNA expression levels of the triplicates were averaged. Following an efficiency-calibrated mathematical model [37], mRNA expression levels for each sample/target gene were averaged and compared to the control group using the REST program [38]. The program calculates relative expression ratios using Eq 1 and employs randomization tests to obtain a level of significance.

$$Ratio=\frac{{\left(E_{target}\right)}^{\Delta {CT}_{target}(control-sample)}}{{\left(E_{ref}\right)}^{\Delta CT_{ref}(control-sample)}}$$ (1)

E_target_ and E_ref_ are the qRT-PCR efficiencies of a target gene and reference gene transcript, respectively; and ΔCT is the difference in control and sample cycle thresholds for the respective gene transcript.

### Fourier Transform Infrared Spectroscopy (FTIR)

Following the experimental methods described above, a third set of experiments was run to analyze collagen’s secondary structure using FTIR. Cells were seeded into twelve dishes (six control and six supplemented with 0.26mM BAPN). After 14 days of differentiation the dishes were rinsed three times with PBS and three times with water. Samples were then directly transferred onto barium fluoride windows and air-dried.

FTIR spectroscopic analysis was performed using a Nicolet iS10 spectrometer (Thermo Fisher Scientific, Waltham, MA). A water vapor background was collected and subtracted from sample data as they were collected. Data were collected from the samples under nitrogen purge at a spectral resolution of 4 cm^-1^. A minimum of three spectra were collected per sample and they were averaged and treated as technical replicates. The amide I and amide II regions (~1400–1800 cm^-1^) were baseline corrected according to published standards [39,40] using OriginPro (OriginLab, Northampton, MA). Underlying peaks within these regions were resolved as Gaussian peaks using second derivative analysis and each spectrum was curvefit using GRAMS/AI (Thermo Fisher Scientific, Waltham, MA). The results from the converged peak fitting were expressed as peak position and percentage area of the peak relative to the area underneath the fitted curve. The investigation focused on peaks corresponding to positions at ~1660cm^-1^ and ~1690cm^-1^, shown to be correlated to mature (HP, hydroxylysylpyridinoline) and immature crosslinks, respectively [31,32,41,42].

### Statistical analysis

All statistical analyses were performed using Statistical Analysis System (SAS Institute, Cary, NC) and a value of p<0.05 was considered significant for all experiments.

To investigate difference in collagen fibril morphology due to the presence of BAPN, D-spacing values measured from each culture dish were averaged to yield a single value. These mean D-spacing values from control (n = 4) and treated (n = 4) samples were then compared using a Mann Whitney *U* test. This nonparametric test was chosen due to the low sample size. To explore differences in the distribution of D-spacing values, the histogram and cumulative distribution function (CDF) of each group was generated. The distributions of the control (n = 217) and treated groups (n = 251) were compared using a k-sample Anderson-Darling (A-D) test as previously described [43].

Differences in mRNA expression between control and BAPN-treated samples were assessed using the REST program in group means for statistical significance by using a Pair Wise Fixed Allocation Random Test. The peak percentage area ratios for the FTIR experiment, were compared between the control and BAPN groups using a Student’s *t*-test.

## Results

### Atomic Force Microscopy

**Fig 2:**
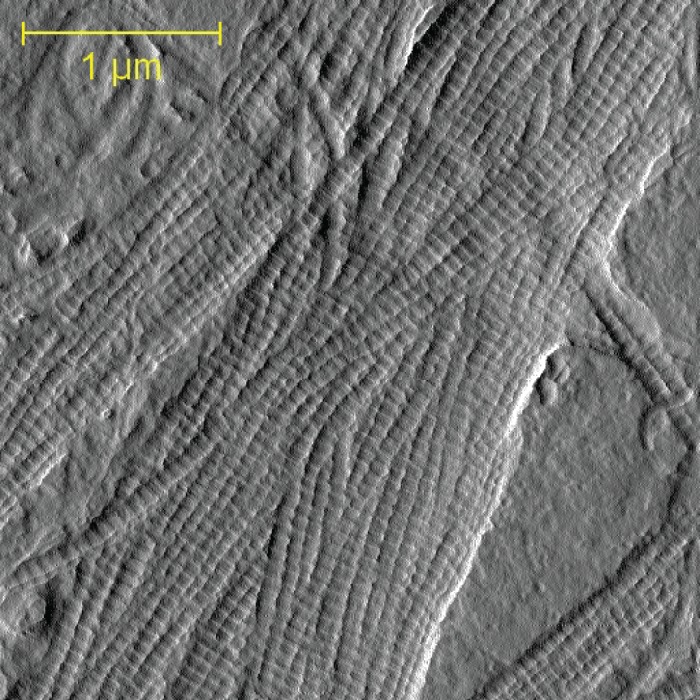
Representative 3.5 μm x 3.5 μm collagen AFM image. AFM images of collagen in its native state were coupled with Fourier transform analysis to measure the periodic fibril D-spacing of collagen synthesized *in vitro* by osteoblasts.

Collagen produced *in vitro* by MC3T3-E1 preosteoblasts was assessed in 60 mm culture dishes. 5 distinct locations were identified and analyzed in each dish using 3.5 μm x 3.5 μm images (Fig 2). A minimum of 10 collagen fibrils were imaged from each of 5 locations, amounting to at least 50 fibrils per dish, and totaling 217 fibril measurements for the control group and 251 for the BAPN-treated group.

**Fig 3:**
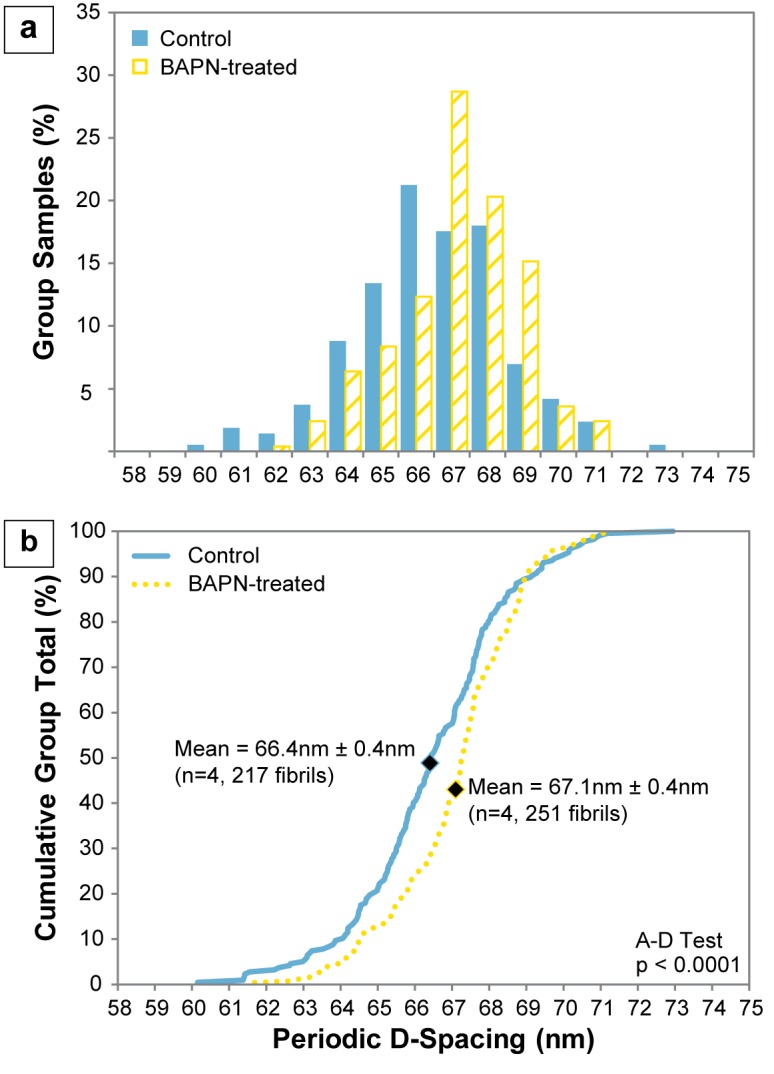
Collagen D-spacing obtained from the D-spacing measurements in each group (n = 4). A clear shift towards higher D-spacing values in the BAPN-treated group is evident in the (a) histogram, (b) cumulative distribution function (CDF), and mean, indicated by the diamond marks on the CDF.

For each dish, fibril D-spacing measurements were pooled to produce the mean D-spacing for that dish, and mean values ranged from 65.8 nm to 66.8 nm for the control group and from 66.6 nm to 67.6 nm for the BAPN-treated group. The mean D-spacing for the 4 samples was 66.4 nm ± 0.4 nm for the control group and 67.1 nm ± 0.4 nm for the BAPN-treated group (p = 0.060) (Fig 3B). When all fibrils in a group were analyzed together, there was a distribution of D-spacing values ranging from 60.2 nm to 72.9 nm for the control group and 61.7 to 71.1 nm for the BAPN-treated group (Fig 3A). These distributions were significantly different from one another with the treated population shifted to higher D-spacing values over most of its range (A-D test, p<0.0001) (Fig 3B).

### Quantitative Reverse Transcription Polymerase Chain Reaction

No significant difference was observed in the mRNA expression of any target gene in the BAPN-treated samples relative to controls (Table 1).

**Table 1:** Fold changes in mRNA expression of BAPN-treated samples relative to controls (n = 5).

Target Gene	Fold Change	Std. Error	95% Confidence Interval	p-value
COL1A1	1.011	0.759–1.452	0.540–1.562	0.966
COL1A2	1.123	0.787–1.447	0.616–1.738	0.471
LOX	1.094	0.655–1.931	0.448–2.569	0.756

### Fourier Transform Infrared Spectroscopy

**Fig 4:**
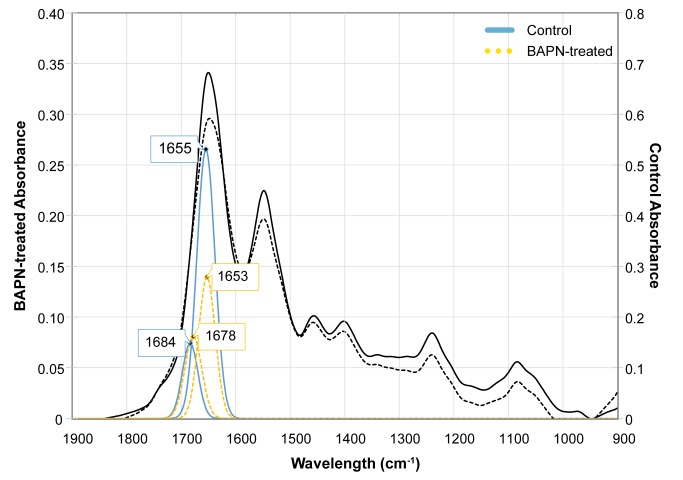
Representative mature and immature crosslink peak fittings underneath the FTIR spectral curve. A decrease in the 1654cm^-1^ peak area is evident in the BAPN-treated sample relative to control. The BAPN-treated and control samples were plotted on different axes to visually highlight this difference. The black solid and dashed lines correspond to the full spectra of control and BAPN-treated samples, respectively.

Peak fitting resulted in consistent peaks around 1654cm^-1^ and 1680cm^-1^ as opposed to the expected 1660cm^-1^ and 1690cm^-1^locations. However; a positive spectral shift of ~10cm^-1^ would result in positions closely matching those reported elsewhere [32,44], therefore peaks found in this study are considered to be representative of HP and immature crosslinks (Fig 4). The spectral shift may be due to interactions with water still present in the samples after air-drying [45–47]. Treatment with BAPN resulted in a decrease in the collagen crosslink ratio driven by a significant reduction in the HP crosslink peak percent area (p<0.05). There was no statistical difference in the percent area of the peak corresponding to immature crosslinks (Table 2).

**Table 2:** Information on underlying FTIR peaks located at ~1660cm^-1^ and ~1690cm^-1^.

		Mean Peak Position (cm^-1^)	Mean Peak Percent Area	Mean Area Ratio
Control	~1660 cm^-1^	1654.3730 ± 0.7289	16.2868 ± 4.1089	3.9068 ± 1.6353
	~1690 cm^-1^	1681.0301 ± 1.5651	4.7963 ± 2.2037	
BAPN	~1660 cm^-1^	1653.4087 ± 0.9959	8.2149 ± 3.4959	1.9865 ± 0.6145
	~1690 cm^-1^	1678.8092 ± 1.0640	4.4880 ± 2.3100	
p-value	~1660 cm^-1^		0.0048	0.0338
	~1690 cm^-1^		0.8177	

## Discussion

It was hypothesized that BAPN treatment would cause partially differentiated MC3T3-E1 cells to synthesize a collagen matrix that was morphologically different from that produced by non-treated cells. Our data indicate that a distribution of D-spacing values, rather than a single value, exists for Type I collagen produced *in vitro* by osteoblasts. The results demonstrate that inhibition of enzymatic crosslinking via BAPN binding of lysyl oxidase causes the D-spacing distribution to shift towards higher values. D-spacing and its distribution provide information on the state and internal structure of collagen and can reflect structural defects in its α chains or changes due to alterations in post-translational collagen synthesis. The inhibition of enzymatic crosslinking via lysyl oxidase inhibition was confirmed by the significant reduction in the mature/immature crosslink ratio and decrease in the peak percent area corresponding to HP, as a result of BAPN treatment. While analysis of D-spacing values showed a significant difference between groups, no difference was found between the control and BAPN-treated groups when comparing gene expression levels. The data showed that treatment of osteoblasts with BAPN does not induce a significant change in expression of any of the genes targeted in this study for their involvement in collagen synthesis. These results contrast those using higher BAPN concentrations in which *COL1A1* was upregulated [32] and *LOX* was downregulated [31,32] with BAPN treatment, and challenges the hypothesis that BAPN would drive upregulation of *LOX* as a response to the decrease in aldehyde formation. Results of this study highlight the effects of post-translational collagen modifications on collagen structure while demonstrating that these changes occurred in the absence of altered collagen or *LOX* gene expression.

The D-spacing distribution has been shown to be capable of reflecting differences in disease states, such as osteoporosis [19] and osteogenesis imperfecta [15,18], as well as tissue types, namely dentin, bone, and tendon [17]. The present study confirms that the D-spacing measure can capture aspects of collagen fibril structure which may relate to the state and internal structure of individual molecules. Data revealed a significant upward shift in the D-spacing distribution between control and treatment groups. The difference in mean D-spacing had a marginally significant p value of 0.06. The low sample size (n = 4) necessitated the use of a nonparametric statistical test. Given the data from the current study, a post-hoc sample size analysis indicates that a sample size of n = 6 would be needed in order to detect a statistical difference between the two groups at 80% power.

The introduction of BAPN, and its binding effect on lysyl oxidase, inhibits the enzymatic crosslinking pathway preventing the formation of aldehyde products. This reduction in aldehyde products has been shown in other osteoblast studies to limit the formation of immature covalent and mature multivalent collagen crosslinks [31,32]. In addition, lysyl oxidase inhibition by a lower in vivo BAPN-fumarate concentration of 0.025mM was found to cause a significant shift in D-spacing in mouse bone from another study completed by our group [48]. After compensating for the added fumarate salt, this concentration corresponds to 0.0137mM BAPN, as compared with the 0.137mM BAPN concentration used in the current study. Given that this tenfold higher 0.137mM BAPN concentration corresponded to five times the half-maximal inhibitory BAPN concentration *in vivo* [49,50], we expected greater lysyl oxidase inhibition to be induced *in vitro*. In other words, the quantitative analysis of collagen synthesized *in vitro* reflects a direct rather than systemic effect of lysyl oxidase inhibition by BAPN. The upward shift in D-spacing distribution seen in the BAPN-treated group of this study suggests that crosslinking may be responsible for compression of fibrils under normal conditions. The same trend was observed in non-mineralized collagen from mouse tail tendon in a study examining the effect of chemical fixation on mouse tail tendon [51]. In contrast, the D-spacing distribution was found to shift toward lower values in mineralized mouse bone collagen with BAPN treatment in vivo [48]. These differences in collagen morphology emphasize the complexity of a crosslink formation process in which *in vivo*/*in vitro* lysyl oxidase inhibition, bone/*de novo* collagen synthesis, and presence/absence of mineral in the collagen matrix all play a role in allowing decompression of the collagen fibril.

Quantitative RT-PCR was used to amplify gene sequences found in RNA isolated from control and BAPN-treated osteoblasts, and to quantify mRNA expression levels. RNA was isolated from osteoblasts after seven days when collagen mRNA expression and synthesis levels were highest [52]. When comparing the two groups, the analysis of fold change in expression showed no difference in *LOX*, *COL1A1*, or *COL1A2* expression. These data indicate that inhibition of the *LOX* enzyme by 0.25mM BAPN-fumarate does not drive a significant upregulation of the *LOX* gene nor does it have a significant effect on the regulation of genes encoding the α1 and α2 helices that form collagen molecules. This supports other BAPN osteoblast studies in which genes encoding *LOX* were not found to be regulated at similar BAPN concentrations [31,32]. This lack of a significant effect suggests that a 0.25mM BAPN-fumarate concentration is too low to induce osteoblasts to compensate for the lack of aldehyde formation and, consequently, collagen matrix formation and stabilization.

FTIR has been used to obtain information about protein secondary structures [39,40], specifically as pertaining to enzymatic collagen crosslinks [45,53]. The use of second derivative methods to locate and fit peaks underlying the FTIR spectral curve has shown a correlation between peaks at 1660 cm^-1^/1690 cm^-1^ and mature/immature crosslinks. [41]. Using this technique, the current study characterized the secondary structure of collagen synthesized *in vitro* by osteoblasts. Peaks were consistently found in the 1654cm^-1^ and 1680cm^-1^ regions, corresponding to mature HP and immature crosslinks, respectively. BAPN treatment resulted in a significant reduction in the HP crosslink peak percent area and decrease in the mature to immature crosslink ratio. The peak percent area of immature crosslinks was not significantly affected. Collectively, these data suggest that a 0.25mM BAPN-fumarate concentration did not inhibit the total amount of available lysyl oxidase enzyme because immature crosslinks were still present. However, the 0.25mM concentration was sufficient to prevent the maturation of divalent immature crosslinks to trivalent HP crosslinks. Results from this study were similar to another in which higher BAPN concentrations also caused an HP crosslink decrease [32] and differed from others which showed no change in the crosslink ratio at comparable BAPN concentrations [31]. These differences in the observed effect of lysyl oxidase inhibition on collagen secondary structure are indicative of a dose-dependent response in enzymatic crosslink formation for both immature and mature crosslinks, particularly relating to HP.

A more thorough investigation of a dose-dependent response to BAPN would elucidate the effect of lysyl oxidase inhibition on collagen gene expression and the single dosage used is considered a limitation of the present study. Consideration of other *LOX* isoforms such as *LOXL1-4* could also provide valuable information in the context of this study given that their expression could differ from that of *LOX*. Future studies will be aimed at assessing these questions and how they relate to osteoblast signaling, collagen production, and post-translational modification.

## Conclusions

In conclusion, collagen synthesized *in vitro* by pre-osteoblastic cells was found to be fibrillar and organized in a manner to produce natural variation in its periodic D-spacing. Although there were no differences in the expression of genes relating to collagen synthesis or enzymatic crosslink initiation, partial lysyl oxidase inhibition at low levels of BAPN still resulted in significant morphological and crosslinking changes in collagen. Collagen fibrils of the BAPN-treated group were found to be morphologically different from those of the control group as seen by the significant increase in the D-spacing distribution of the BAPN-treated collagen fibrils. In addition, the ratio of mature to immature crosslinks was found to decrease with BAPN treatment, associated with a reduction in peak percent area of mature crosslink HP. These findings were made possible by *in vitro* treatment with BAPN and analysis of collagen synthesized entirely under direct inhibition of lysyl oxidase and, thus, enzymatic crosslink formation.
